# Concomitant Reversible Cerebral Vasoconstriction and Hyperperfusion Syndromes Following Carotid Endarterectomy

**DOI:** 10.7759/cureus.8541

**Published:** 2020-06-09

**Authors:** Ali Tayebi Meybodi, Amit Singla, ZeGuang Ren, Shih Liu

**Affiliations:** 1 Neurosurgery, Rutgers New Jersey Medical School, Newark, USA; 2 Neurosurgery and Brain Repair, University of South Florida, Tampa, USA

**Keywords:** carotid stenting, carotid stenosis, cerebral hyperperfusion syndrome, intracranial hemorrhage, reversible cerebral vasoconstriction syndrome

## Abstract

Cerebral hyperperfusion syndrome (CHS) and reversible cerebral vasoconstriction syndrome (RCVS) are two syndromes characterized by disordered vascular autoregulatory mechanisms of brain. These may be seen after carotid endarterectomy (CEA). We present a patient who developed both syndromes after CEA which is a rare occurrence.

## Introduction

Cerebral hyperperfusion syndrome (CHS) is a rare but well-known complication after major cerebral revascularization procedures including carotid endarterectomy (CEA) and carotid artery angioplasty/stenting (CAS) [1-2]. Clinically, CHS presents with any or various combinations of (1) throbbing retro-orbital and/or frontotemporal headache, (2) seizures, (3) focal neurological deficits in the absence of thromboembolic events, and (4) intracranial hemorrhage (ICH) [2-3]. The pathophysiological hallmark of CHS is a severely increased cerebral blood flow (CBF) in a previously hypoperfused brain with maximally dilated vessels that are unable to manage the increased CBF due to a loss of autoregulatory mechanisms [3-5].

On the other hand, the reversible cerebral vasoconstriction syndrome (RCVS) is another known complication after CEA or CAS among many other causes recognized for RCVS [6]. This syndrome is associated with repeated episodes of thunderclap headaches, seizures, nonthromboembolic focal neurological deficits, and various forms of ICH. Importantly, RCVS is associated with diffuse segmental spasm of the cerebral arteries [6-8]. Similar to CHS, this syndrome represents a dysfunctional autoregulatory capacity of the previously hypoperfused cerebral vascular bed that undergoes severe vasospasm following CEA or CAS [6-8]. Both CHS and RCVS can be associated with ICH after CEA or CAS [7, 9-10]. However, to the best of our knowledge, there are only two reports of concomitant occurrence of CHS and RCVS in the post-CEA setting [11-12]. The present case is the third report of this dual complication occurring in a patient undergoing CEA.

## Case presentation

**Figure 1:**
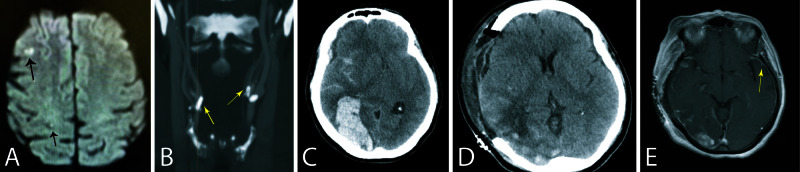
Pre- and post-operative brain images. A: Pre-operative diffusion weighted brain MRI showing previous punctate infarcts in the right middle cerebral artery territory (arrows). B: Cervical CT angiography showing bilateral dense calcified plaques at the carotid bulb and severe right internal carotid artery stenosis (arrows). C: Post-endarterectomy brain CT scan showing a large temporo-occipital intraparenchymal hemorrhage, subarachnoid, and subdural hemorrhage. D: Brain CT scan after decompressive craniectomy and clot evacuation. E: Post-endarterectomy gadolinium enhanced brain MRI showing clot evacuation and diffuse leptomeningeal enhancement on postdecompression day 6 (arrow).

A 59-year-old female with a history of hypertension presented to an outside hospital with symptoms concerning for transient ischemic attacks affecting the right middle cerebral artery (MCA) territory causing brief (<15 min) episodes of left hemiparesis. Imaging showed previous infarcts in the right frontal cortex (Figure 1A), as well as bilateral dense calcified plaques at the carotid bulb and severe right internal carotid artery (ICA) stenosis (>90%) (Figure 1B). She underwent a right CEA and was discharged home on post-op day 2. On post-op day 4, she presented to the outside hospital with acute mental status change. Neurological examination revealed 4 mm reactive pupils, irregular respirations, and bilateral flexor posturing in response to noxious stimuli. She was intubated immediately. Imaging showed a large right temporo-parieto-occipital hemorrhage with about 20 mm midline shift, adjacent cortical subarachnoid hemorrhage (SAH), and small right frontotemporal subdural hemorrhage (SDH) (Figure 1C). These signs were concerning post-CEA CHS. She was intubated and referred to our hospital where she underwent an emergent decompressive craniectomy and clot evacuation (Figure 1D,E). The bone flap was not replaced after craniectomy.

**Figure 2:**
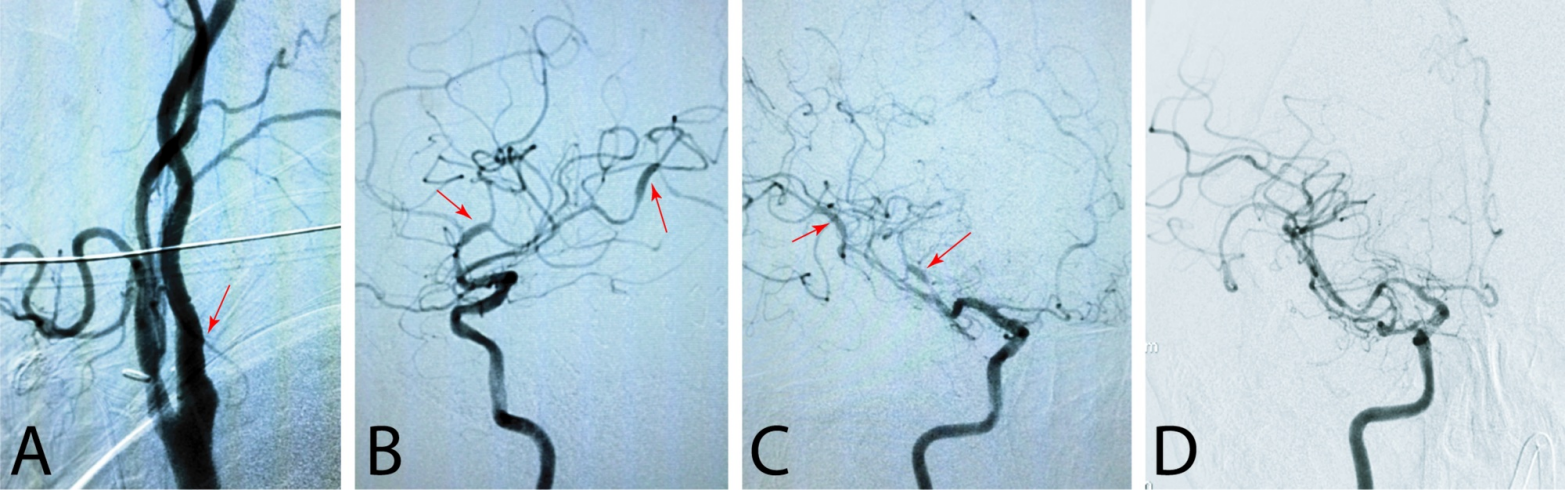
Cerebral angiogram. A: Catheter angiogram images showing resolution of right internal carotid artery stenosis after endarterectomy. Arrow points to the internal carotid artery. Anteroposterior (B) and oblique (C) right middle cerebral artery angiograms after decompressive craniectomy and clot evacuation showing “sausage-on-string” sign of reversible cerebral vasoconstriction syndrome (RCVS) (arrows). D: after two weeks from clot evacuation, cerebral angiogram shows almost complete resolution of segmental arterial spasm.

The patient improved clinically on post-craniectomy day 1 having purposeful extremity movements and following commands occasionally. However, she started to have seizure-like activity (spastic jerks in all extremities) lasting for hours, although her electroencephalogram (EEG) was negative. Levetiracetam and lacosamide were started. Due to the occurrence of post-CEA ICH and questionable head CT angiography concerning for vasculitis, cerebral catheter angiography was performed on post-craniectomy day 7. The angiogram demonstrated a patent right ICA (Figure 2A) and diffuse segmental constrictions and dilatations of the MCA concerning for segmental vasospasm while vasculitis could not be ruled out (Figure 2B,C). Therefore, steroids were started to cover for any vasculitic pathology. However, a vasculitis work-up was negative. A repeat catheter angiogram 10 days later demonstrated almost complete resolution of the segmental constrictions and dilatations favoring a reversible vasospasm (i.e., RCVS) over vasculitis (Figure 2D,E). The MRI brain did not show any new infarction. The family refused any further surgeries. She was discharged to a nursing home under hospice care. At the time of her discharge she was able to follow commands and utter a few words. She had a sunken right skin flap at the site of her craniectomy. She was lost to follow up.

## Discussion

We report a patient with post-CEA CHS presenting with ICH whose clinical course was complicated with RCVS. Fitas et al. reported a 55-year-old-female with post-CEA imaging signs such as cortical edema and focal cortical SAH (compatible with early post-operative CHS) which was later complicated by increasing Lindegaard ratios in serial transcranial Doppler studies (over the course of 10 days) compatible with RCVS [12]. Also, Asuzu et al. reported a 54-year-old female with post-CEA multifocal intraparenchymal hemorrhages as well as SDH and SAH whose clinical course was complicated by post-CEA nonthromboembolic infarcts caused by RCVS confirmed by catheter angiography [11]. We believe that these two reports describe patients with symptoms compatible with initially evolving post-CEA CHS that was later complicated by RCVS. To the best of our knowledge, we are reporting the third case with a similar clinical course.

CHS after CEA

Cerebral hyperperfusion syndrome is a well-known complication after carotid artery revascularization procedures including CEA and CAS [3, 9]. This complication was first described by Sundt et al. who showed that dramatic (>200%) increase in CBF can occur after CEA and later verified by others [13]. It is postulated that following long-standing carotid stenosis, the vasculature of the hypoperfused brain develops maximal vasodilation to in an attempt to maximize CBF. Of note, the degree of carotid stenosis required to cause maximal brain vasodilation is not clear. However, it is conceivable that the stenosis should be hemodynamically significant. This long-standing vasodilation of the cerebral circulation leads to a loss of autoregulation capacity [3, 13]. Elimination of the high-grade stenosis in the carotid artery causes a blood flow surge to the brain with the “paralyzed” vascular bed of the brain that remains undefended against this “blood flood” (i.e., increased CBF). This process leads to the classic CHS characterized by headaches, vomiting, cerebral edema which may cause seizures, and focal neurological deficit(s). The pathophysiology of CHS after CEA is similar to the “normal perfusion pressure breakthrough” phenomenon described by Spetzler et al. after removal of cerebral arteriovenous malformations [14]. In extreme cases, increased blood flow may cause intraparenchymal hemorrhage which is usually large and has a poor prognosis, although intraventricular hemorrhage (IVH) and SAH have also been reported [15]. Occurrence of a large ICH in our patient after CEA (or CAS) is a rare (overall incidence ~0.5%) but compelling sign of CHS. Also, diffuse leptomeningeal enhancement has been described as another sign of CHS that may reflect the increased blood flow in the cranial arteries (Figure 1E) [16].

Angiographic findings reported for CHS include: (1) extravasating contrast material in distal vascular bed of the brain, and (2) arteriovenous shunting signs with early draining vein(s) and capillary blush during the CHS phase which fade after resolution of CHS [17].

Management of CHS is generally towards aggressive control of blood pressure keeping it within normal limits if not lower, in addition to symptomatic treatment for seizures and brain edema [3]. Beta-blockers (especially the mixed alpha- and beta-blocker labetalol) are proposed because they do not increase the CBF. Use of vasodilators such as nitrates, calcium-channel blockers, and hydralazine is clearly risky in the setting of CHS because of higher chance of vasodilation. Additionally, angiotensin converting enzyme inhibitors may theoretically increase CBF. In cases of ICH, reversal of anti-coagulation and optimizing platelet function are essential. When massive intraparenchymal hemorrhage complicates the course of CHS (such as the presented case), surgical evacuation may be a choice as shown in the presented case. 

RCVS after CEA

Reversible cerebral vasoconstriction syndrome is a relatively newly described entity after CEA. Although episodic vasoconstriction has been reported before, Call and Fleming were the first to systematically describe this syndrome in 1988 [18]. It is clinically characterized by episodes of thunderclap headache, with or without seizures and focal neurological deficits associated with reversible segmental vasoconstriction of cerebral arteries. Headache is seen in most patients but may be absent [6]. RCVS is usually seen in middle aged people (although all ages may be affected) and affects women more than men [6, 8]. It usually happens after a trigger event such as post-partum state, use of vasoactive drugs, catecholamine-secreting tumors, and use of immunosuppressant drugs or blood products. Several cases have been reported after CEA or CAS during which a post-procedural accentuated vasoconstrictive response is seen after the first reported case by Brick et al. in 1990 [19].

Angiographically, the cerebral vessels show segmental spasm which is diffuse and bilateral and affects both anterior and posterior circulations [8]. Typically, the “sausage-on-string” sign is seen on angiography (Figure 2B,C). The angiographic appearance may be mistaken for “vasculitis.” It may also be similar to post-SAH vasospasm. However, following SAH, vasospasm usually causes steady nonsegmental vasoconstriction on angiography (a finding that was not seen in our patient, lowering the likelihood of the vasoconstriction to be a sequel of the existing SAH). 

Reversible cerebral vasoconstriction syndrome may be associated with ischemic or hemorrhagic stroke. Hemorrhagic sequelae include different forms of ICH, namely, SDH, SAH, and intraparenchymal hemorrhage [20]. The most common association is nonaneurysmal SAH which is frequently found in the cerebral convexities and superficial sulci and is believed to be originating from the small pial vessels [6]. The presented case shows all the three ICH subtypes from which the SAH and SDH may be attributed to RCVS, although the large IPH is more consistent with post-CEA CHS.

Treatment of RCVS is usually supportive. If present, any existing vasoactive medications should be discontinued. Calcium channel blockers have been suggested to relieve the vasoconstriction, although they may not necessarily prevent cerebral vasoconstriction or ICH [6]. Intra-arterial calcium channel blockers may be needed (sometimes repeatedly) for treatment of symptomatic RCVS. They may be also useful in the post-CEA setting as these agents can control hypertension. However, they may cause hypotension which can be harmful in the setting of diffuse cerebral vasoconstriction. 

The Management Dilemma of Co-existing CHS and RCVS

Reversible cerebral vasoconstriction syndrome and CHS represent two opposite ends of the pathophysiological spectrum of post-CEA/CAS cerebral dysautoregulation. On one end of the spectrum, the paralyzed arterioles of the brain cannot resist the suddenly increased CBF via proper reciprocal vasoconstriction (CHS). On the other end, diseased autoregulation of the brain leads to exaggerated vasoconstriction after CEA or CAS that may result in RCVS. These phenomena were both seen in the presented case. This constellation of pathophysiological phenomena creates a management challenge. Treatment of CHS requires lowering blood pressure control and avoiding vasodilator medications, whereas treatment of RCVS requires vasodilator therapy, and possibly controlled hypertensive therapy to treat strokes (if present). 

There is no standard therapy for co-existing CHS and RCVS. We suggest meticulous work up of candidates for CEA/CAS to identify patients at increased risk for disordered cerebral autoregulation (see above, post-CEA CHS). These patients should be carefully followed after revascularization and be treated in a timely fashion to prevent the devastating complications of stroke and/or ICH. When evidence exists to support the coexistence of both syndromes, we suggest cautiously treating the prevailing syndrome (CHS versus RCVS) which is creating the symptoms while avoiding overtreatment (that may cause the other syndrome to become symptomatic) and staying vigilant for the symptoms of the other syndrome. These patients may be followed with daily transcranial Doppler or other studies to detect signs of dysfunctional autoregulation [12].

## Conclusions

Reversible cerebral vasoconstriction syndrome and CHS represent the two ends of the dysautoregulation spectrum in post-CEA patients. We report a rare case of concomitant CHS and RCVS after CAE. Proper identification of high-risk patient may warrant more diagnostic tests to detect the signs of dysautoregulation in post-CEA patients to prevent poor outcomes from these syndromes.
